# Serum and Peritoneal Fluid Levels of Vascular Endothelial Growth Factor in Women with Endometriosis

**Published:** 2013-07-31

**Authors:** Maryam Kianpour, Mehdi Nematbakhsh, Sayad Mehdi Ahmadi, Mehrangiz Jafarzadeh, Masomeh Hajjarian, Zahra Pezeshki, Tahereh Safari, Fatemeh Eshraghi-Jazi

**Affiliations:** 1 Nursing and Midwifery Care Research Centre, Department of Midwifery, Faculty of Nursing and Midwifery, Isfahan University of Medical Sciences, Isfahan, Iran; 2 Water and Electrolytes Research Centre, Department of Physiology, Kidney Diseases Research Center, Isfahan University of Medical Sciences, Isfahan, Iran; 3 Isfahan Fertility and Infertility Centre, Isfahan, Iran

**Keywords:** Endometriosis, Vascular Endothelial Growth Factor, Peritoneal Fluid

## Abstract

**Background:**

Endometriosis is known as one of the most common disease in women of reproductive age. Due to important role of vascular endothelial growth factor (VEGF) in neo-vascularization for the implantation of endometrial cell, and also presence of different studies reported VEGF level in the serum and peritoneal fluid (PF) in endometriosis patients, this study was designed to determine the serum and PF levels of VEGF in endometriosis patients, and to compare with normal subjects.

**Materials and Methods:**

In this descriptive study, 179 women subjected to laparoscopy for the evaluation of infertility or pelvic pain were allocated into the following two groups: group I: different types of endometriosis patients (n=90) and group II: non-endometriosis patients (n=89). The PF from pelvis and venous blood samples were obtained. The VEGF concentration of the serum and PF were measured using enzyme immunoassay kit and were compared using t test.

**Results:**

The level of VEGF in serum was significantly less than that in PF in both groups (p=0.00). However, endometriosis patients had significantly higher level of VEGF in peritoneal fluid than non-endometriosis patients (p=0.043).

**Conclusion:**

According to our findings, endometriosis is not associated with change in the level of circulating VEGF.

## Introduction

Angiogenesis is identified as new capillaries being formed from pre-existing blood vessels. This phenomenon involves the interaction of some of the closely synchronized molecules including vascular endothelial growth factor (VEGF) (1, 2). VEGF, as a kind of mitogen, is a major promoter of angiogenesis in pathological and physiological settings, and is also considered as a survival factor for endothelial cells (3). Furthermore, through vascular leakage and mobilizing leukocytes, VEGF, as a strong vascular permeability factor, promotes inflammatory process (4, 5).

Endometriosis is known as one of the most common disease in women of reproductive age. Despite the current controversy regarding the pathophysiology of this disease (6), Sampson’s theory explains the existence of endometrial cells in the peritoneal cavity by retrograde menstruation. Several factors, such as increased of inflammatory activity in the peritoneal fluid (PF), angiogenesis and up-regulating of pro-inflammatory cytokines may facilitate the pathogenesis of endometriosis, which is assumed to be a complex process (7, 8).

There is a controversy among the literatures about the possible variation of VEGF in serum and in PF of endometriosis patients (9-20). Some of the published data indicated the increased level of VEGF in serum (9, 13, 19) and in PF (10, 11, 13) of endometriosis patients. However, several others studies reported no change of VEGF level in serum (15-18) and in PF (12, 15) of endometriosis patients. On the other hand, there have been many theories explaining the etiology, but the most common accepted one is shedding of the endometrium following retrograde menstruation. In endometriosis, neo-vascularization is essential for the successful implantation of endometrial cells in ectopic sites (2). VEGF is part of a heparin-binding protein family (21), and the induction of endometrial cell proliferation is functioned by VEGF (4, 22), so VEGF was considered essential factor in uterine angiogenesis (23).

Most of published data related to VEGF variation in endometriosis were obtained from small sample size patients, and accordingly, we attempted to consider this inconsistency in a large number of patients referred to a clinic during three years. Therefore, we measured the level of VEGF in serum and in PF of endometriosis patients and compared with normal subjects.

## Materials and Methods

### Patients

This descriptive study was approved by Ethical Committee of Isfahan University of Medical Sciences. During years 2009-2011, a total of 392 patients subjected to laparoscopy for the evaluation of infertility or pelvic pain at the Isfahan Fertility and Infertility Center were considered. The medical record of each patient was exactly reviewed by an expert gynecologist in order to exclude those patients with hypertension, coronary arterial diseases, diabetes, renal diseases, active pelvic inflammatory disease or polycystic ovarian syndrome. Finally, 179 patients were assigned for this study. This sample size was selected based on our pilot study. After laparoscopy, the patients were allocated into the following two groups: group I: different types of endometriosis patients (n=90) and group II: non-endometriosis patients (n=89). The official informed consent was obtained for all subjects.

### Collection of serum and peritoneal fluid

The venous blood samples were obtained from all patients before induction of anesthesia for laparoscopy procedure. The blood samples were centrifuged, and the serums were stored at -20˚C until measurement. In addition, the PF samples were collected from pelvis before any manipulation. The bloody fluids were excluded. The PF samples were also centrifuged and the supernatant were stored at -20˚C until measurement.

### VEGF measurement

The serum and peritoneal levels of VEGF were measured using enzyme immunoassay kit (Immuno- Biological Laboratory Co., Japan). Briefly, the kit is a solid phase sandwich enzyme linked immunosorbent assay (ELISA) using specific polyclonal and monoclonal antibodies, while the coloring agent was tetra methyl benzidine (TMB). The sample (100 μl) was put in pre coated plate (Anti-Human VEGF (16F1) Mouse IgG M0Ab Affinity Purify (Immuno-Biological Laboratories Co., Ltd, Japan), then washed after incubating for 60 minutes in 37˚C. Afterwards, the labeled antibody (HRP conjugated Anti-Human VEGF Rabbit IgG Fab’ Affinity Purify (Immuno-Biological Laboratories Co., Ltd, Japan), was added, incubated and washed again. Finally, coloring agent was added, followed by stop solution, whereas the absorbance was determined at 450 nm by plate reader. The VEGF concentration was determined using standard curve.

### Statistical analyses

Data was expressed as mean ± SEM. Unpaired t test was applied to compare the parameters between the groups, while paired t test was applied to compare the parameters within the groups. The value of p<0.05 was considered statistically significant.

## Results

**Fig 1:**
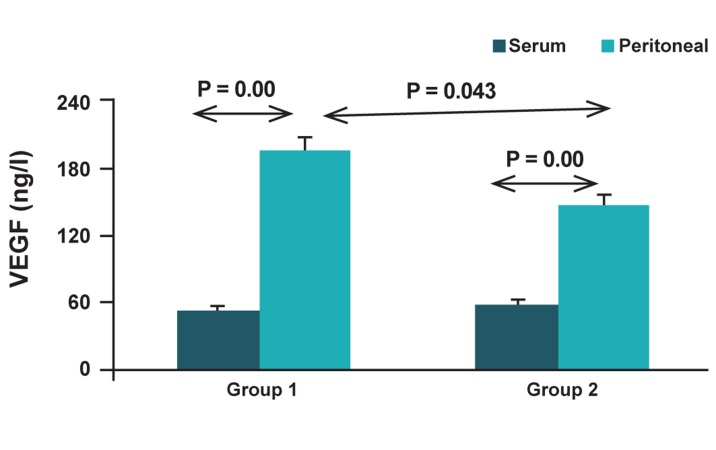
The VEGF level in serum and peritoneal fluid from the patients with endometriosis (group I) and control subjects (group II).

The average age of groups I and II are 28.9 (range: 19-44) and 30.2 (range: 24-42), respectively, while there was no statistically significant difference between two groups. In this study, 166 women were in proliferative phase, and 13 women were in secretory phase. The obtained data for VEGF level in serum and in PF from group I (the patients with endometriosis) and group 2 (control) are demonstrated in figure 1. The VEGF level in serum was significantly less than that in PF in both groups (p=0.00). However no significant difference in serum level of VEGF was detected between the two groups, but the result indicates that group I had significantly higher level of VEGF in peritoneal fluid in comparison to group II (p=0.043).

## Discussion

Endometriosis, as a gynecologic disease, has been subjected to many research in order to find the exact cause of infertility related to this disorder (2, 6-8, 11, 14, 18, 19, 24, 25). The main objective of this study was to compare the serum and peritoneal levels of VEGF in endometriosis patients and control subjects. We found that the level of VEGF in PF were higher than that in serum in both groups. In addition, the peritoneal level of VEGF in endometriosis was increased significantly when compared with control group.

In some studies, the serum level of VEGF in endometriosis was reported differently compared to our obtained result. Their findings showed higher levels of VEGF in the serum of patients with endometriosis compared to specific control group (9, 10); in addition, no significant difference was detected in endometriosis patients in the luteal phase (which may affect the variation of VEGF) with different control group undergoing surgery for several different indications (tubal ligation, hysterectomy or diagnostic laparoscopy) (16). In other study, the VEGF level in patients in different phases of the menstrual cycle was compared with false-positive cases (patients who were suspected to have endometriosis), and no statistically difference was detected even when the phase of the cycle was taken into account (12). One of the advantages of our data was the specific control group consisting of infertile women with no evidence of endometriosis in their laparoscopic examination. It seems that this control group was more reliable to compare with endometriosis patients. Similar to the other study (12, 16), we also included all stages of endometriosis in our study group (group I).

Endometriosis is a chronic disease associated with a general inflammatory response in peritoneal cavity. Evidence shows that immunological factors (26) and angiogenesis play a decisive role in the pathogenesis of endometriosis (13, 27), and there is an increase in number, activity and secretion of peritoneal macrophages of endometriosis cases (28, 29). In women with endometriosis, the function of peritoneal macrophages, killer cells and lymphocytes are so considerable. Moreover, growth factors and inflammatory mediators in the peritoneal fluid are mainly produced by peritoneal macrophages, and peritoneal leukocytes, are modified in endometriosis (13). It seems the peritoneal macrophages activate (29) endometrial cells (30), and all neutrophils (31) are also able to synthesize and secret VEGF (10, 27). VEGF promotes inflammatory process through vascular leakage and leukocytes accumulation (32). Therefore, a local increase of the VEGF in the PF (not in serum) might be due to an increase of the macrophage secretary products, which is found to be a major source of VEGF in endometriosis (10).

Also, VEGF is one of those molecules attributed to the growth and maintenance of angiogenesis (4, 12, 22, 26, 33) and vascular permeability (10, 12, 33). Accordingly, it can be a main factor in physiologic angiogenesis in endometrium (9, 28). It has been shown higher angiogenic activity of PF in woman suffering from endometriosis in comparison to those not suffering from this disease (11). It was observed that there is an increase in angiogenesis around the peritoneal explants, followed by an increase in angiogenic activity in the PF of endometriosis women (11, 34). Vascularization within and around the tissue have been pronounced by active endometriotic explants, caused by the process of angiogenesis (35). Surprisingly, both peritoneal macrophages and deep endometriotic lesions produce VEGF in large amounts in PF (36). Therefore, the ability of endometriosis lesions in producing VEGF is another reason for an increase in PF level in endometriosis women (30).

## Conclusion

According to our data, it seems that angiogenic activity may increase by the elevated level of VEGF in the PF of endometriosis patients. This elevated level of VEGF possibly promotes neovascularization within the peritoneal environment. It seems, this disease is only associated with pelvic inflammation, while is not related to the change of VEGF level in circulation.
